# The diagnostic value of ictal SPECT—A retrospective, semiquantitative monocenter study

**DOI:** 10.1002/epi4.12694

**Published:** 2023-01-30

**Authors:** Freya Schulte, Felix Bitzer, Florian Christoph Gärtner, Tobias Bauer, Randi von Wrede, Tobias Baumgartner, Attila Rácz, Valeri Borger, Tim von Oertzen, Hartmut Vatter, Markus Essler, Rainer Surges, Theodor Rüber

**Affiliations:** ^1^ Department of Epileptology University Hospital Bonn Bonn Germany; ^2^ Department of Nuclear Medicine University Hospital Bonn Bonn Germany; ^3^ Department of Neurosurgery University Hospital Bonn Bonn Germany; ^4^ Department of Neurology 1, Neuromed Campus Kepler University Hospital, Johannes Kepler University Linz Austria

**Keywords:** epilepsy, neuroimaging, perfusion, presurgical evaluation, seizure onset zone

## Abstract

**Objective:**

Ictal single photon emission computed tomography (SPECT) can be used as an advanced diagnostic modality to detect the seizure onset zone in the presurgical evaluation of people with epilepsy. In addition to visual assessment (VSA) of ictal and interictal SPECT images, postprocessing methods such as ictal‐interictal SPECT analysis using SPM (ISAS) can visualize regional ictal blood flow differences. We aimed to evaluate and differentiate the diagnostic value of VSA and ISAS in the Bonn cohort.

**Methods:**

We included 161 people with epilepsy who underwent presurgical evaluation at the University Hospital Bonn between 2008 and 2020 and received ictal and interictal SPECT and ISAS. We retrospectively assigned SPECT findings to one of five categories according to their degree of concordance with the clinical focus hypothesis.

**Results:**

Seizure onset zones could be identified more likely on a sublobar concordance level by ISAS than by VSA (31% vs. 19% of cases; OR = 1.88; 95% Cl [1.04, 3.42]; *P* = 0.03). Both VSA and ISAS more often localized a temporal seizure onset zone than an extratemporal one. Neither VSA nor ISAS findings were predicted by the latency between seizure onset and tracer injection (*P* = 0.75). In people who underwent successful epilepsy surgery, VSA and ISAS indicated the correct resection site in 54% of individuals, while MRI and EEG showed the correct resection localization in 96% and 33% of individuals, respectively. It was more likely to become seizure‐free after epilepsy surgery if ISAS or VSA had been successful. There was no MR‐negative case with successful surgery, indicating that ictal SPECT is more useful for confirmation than for localization.

**Significance:**

The results of the most extensive clinical study of ictal SPECT to date allow an assessment of the diagnostic value of this elaborate examination and emphasize the importance of postprocessing routines.

**Key points**

- Computational postprocessing of ictal SPECT increases the chance to localize the seizure onset zone by a factor of 1.88.
- Ictal SPECT is more useful in temporal as compared to extratemporal epilepsy.
- Ictal SPECT is worse than MRI, but better than interictal and ictal EEG, when reduced to its ability to localize the seizure onset zone.
- The odds of becoming seizure free after epilepsy surgery were higher after successful as compared to unsuccessful ictal SPECT.
- Ictal SPECT is better in confirming the seizure onset zone in MR‐positive cases, than in localizing it in MR‐negative ones.

## 1 INTRODUCTION

The localization of the seizure onset zone (SOZ) in people with pharmacoresistant epilepsy undergoing presurgical diagnostics often remains a major challenge. The standard presurgical evaluation includes electroencephalography (EEG) examinations, analysis of semiology, neuropsychological testing, and magnetic resonance imaging (MRI).^1^ Ictal single photon emission computed tomography (SPECT) can be used as an advanced diagnostic modality to detect the SOZ.^2^, ^3^, ^4^ However, including video and EEG monitoring of epileptic seizures and possible multiple injections of the tracer, ictal SPECT is an expensive and time‐consuming imaging modality^5^, ^6^ in which individuals are exposed to an average radiation dose of 6.8 millisievert (mSv).^7^ The added value of SPECT is seen particularly in people with extratemporal epilepsies and those in whom no potentially epileptogenic lesion could be detected in MRI (MR‐negative).^2^

While the rest of epilepsy imaging is centered around the potentially epileptogenic lesion, SPECT is unique in the presurgical workup, as it is the only imaging modality geared towards visualizing correlates of an epileptic seizure.^8^ During an epileptic seizure, perfusion to the SOZ is increased by up to 300%, in response to the local neuronal hyperactivity.^9^, ^10^ To visualize this hyperperfusion, a radioactive tracer (often ^99m^ Tc‐HMPAO) is injected during a seizure. The uptake of the tracer is proportional to the regional cerebral blood flow due to a high first‐pass effect, and it remains fixated in the brain tissue for several hours after injection.^9^ SPECT imaging can therefore be performed after postictal recovery of the individual and it represents a “snapshot”‐image of the ictal regional cerebral blood flow. Additionally, an interictal image is acquired, to visualize the baseline regional cerebral blood flow of the individual.

**FIGURE 1:**
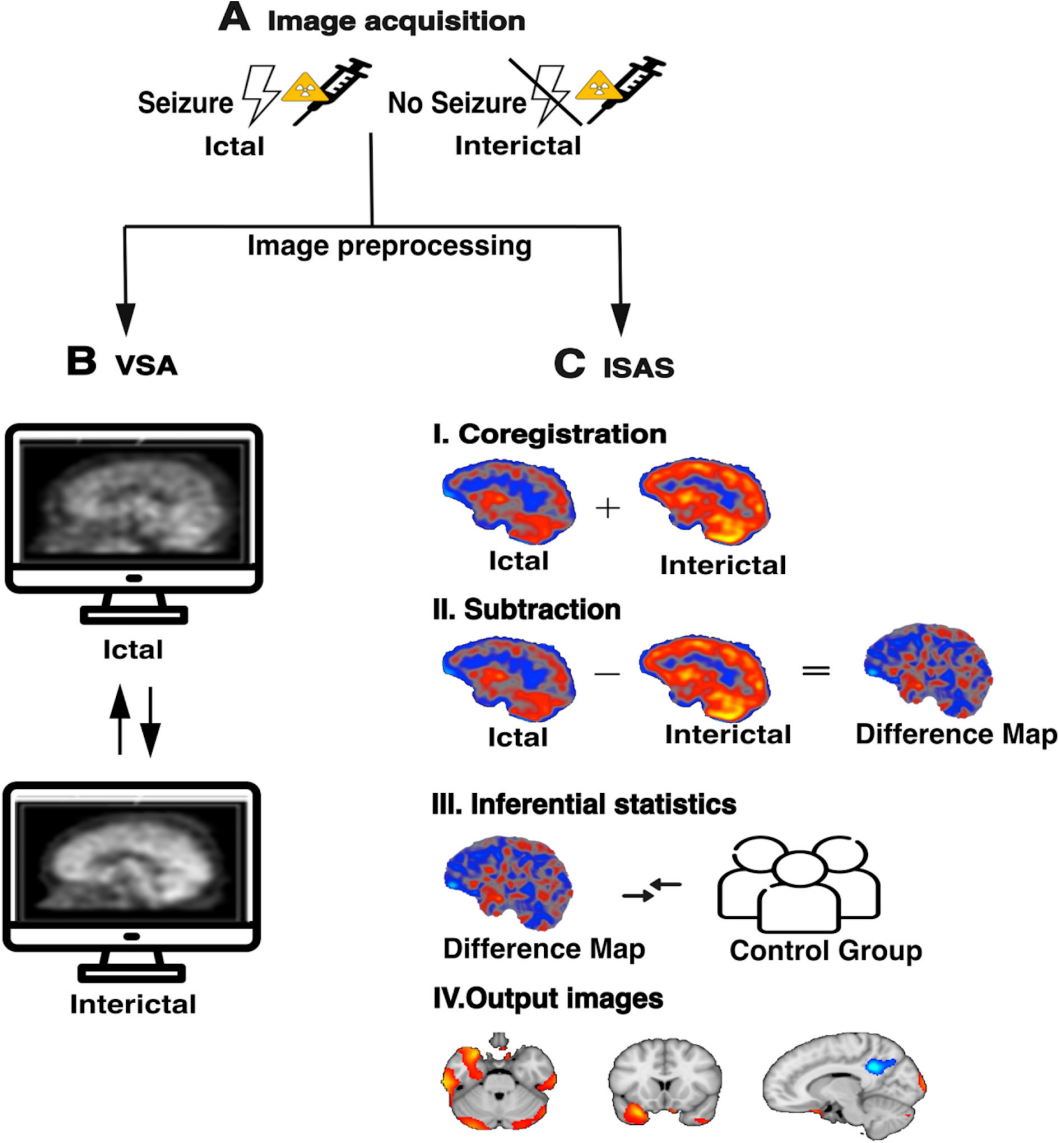
Schematic of image acquisition (A), visual SPECT assessment (B) and Ictal‐Interictal SPECT Analysis by SPM (C).

Differences between the ictal and interictal images can then be identified through a simple side‐by‐side visual SPECT assessment (VSA) of the scans. For further analysis of ictal SPECT beyond visual inspection, several different postprocessing software packages have been developed to identify pathological alterations in postictal perfusion. One of the most popular methods is Ictal‐Interictal SPECT Analysis by SPM (ISAS), introduced in 2002 by Chang and colleagues.^11^ The ISAS tool assesses the statistical significance of the regional cerebral blood flow differences on a voxel‐by‐voxel basis and maps the results to a structural MRI volume of the subject. Both hyperperfusions and hypoperfusions are mapped, although the relevance of hypoperfusions remains questionable.^11^ A variance map of physiological scan‐to‐scan variations of 14 paired healthy control images^11^, ^12^, ^13^ needed for inferential statistics is included (see Figure 1).

Several studies have demonstrated the superiority of these quantitative methods over side‐by‐side visual inspection.^14^, ^15^, ^16^ In quantitative methods, the subjective nature of visual inspection is eliminated and subtle ictal‐interictal difference can be detected by quantitative analysis. Additionally, the localization precision is improved by mapping the results to the structural MRI image of the subject.^5^, ^6^, ^17^, ^18^ Most SPECT studies focus on the concordance of SPECT results and the resected focus in a small cohort of operated individuals,^19^, ^20^, ^21^, ^22^ but only few studies have compared VSA and ISAS in a large, unselected cohort.

In this study, we associated results of VSA and ISAS with the resection site and postsurgery outcome in individuals who underwent surgery and with the clinical focus hypothesis in individuals who did not undergo surgery. The aim of this study was to calculate sensitivity of VSA and ISAS, estimate their diagnostic value and evaluate differences between these two methods and other diagnostic modalities in the Bonn cohort.

## 2 MATERIALS AND METHODS

### 2.1 Study group

Inclusion criteria were ictal and postictal SPECT imaging followed by ISAS postprocessing as part of a presurgical evaluation at the Department of Epileptology at the University Hospital Bonn between 2008 and 2020. In case individuals underwent SPECT examination twice, we only included data from the more recent SPECT acquisition in our analyses. The study was approved by the IRB of the University Hospital Bonn.

### 2.2 Clinical characteristics

Clinical characteristics were obtained from the clinical records and included: Interictal and ictal EEG, report and interpretation of semiology, neuropsychological test report, MRI results, FDG‐PET results, VSA results, ISAS results, seizure–injection‐latency, anatomical location of implanted electrodes (if invasive EEG monitoring had been performed), anatomical location of surgical resection and histology. The clinical focus hypothesis had been established by a multidisciplinary team of expert physicians based on concordance of results of localizing diagnostic modalities as part of the clinical routine and was extracted from the individuals' records for our analysis. Seizure freedom was assessed both after 1 year and at the latest possible timepoint. For our analyses, we chose the latest possible timepoint/clinical follow‐up (median 32 months post op, range [2,136]).

### 2.3 Image preprocessing

VSA means side‐by‐side comparison of the ictal and interictal SPECT images by an experienced nuclear physician. ISAS was performed following the ISAS protocol as first described by Chang and colleagues.^11^

### 2.4 Categorization of SPECT outcome

**FIGURE 2:**
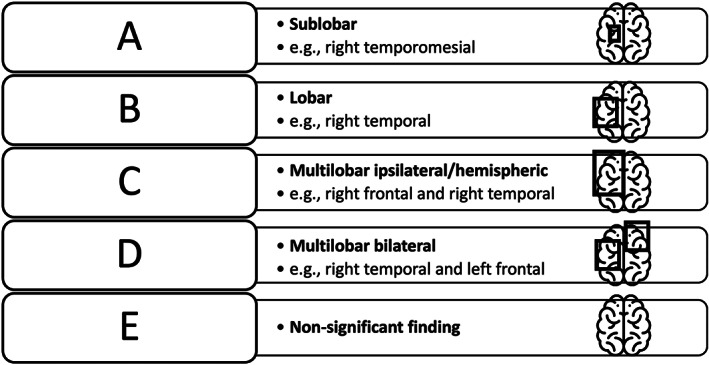
Classification of intermodal coherence between visual SPECT assessment (VSA)/ictal‐interictal SPECT Analyses by SPM (ISAS) and the clinical focus hypothesis in alphabetical order and with decreasing accuracy of the findings.

The alignment between the VSA‐/ISAS‐output and the clinical focus hypothesis was analyzed following the categorization by El Tahry and colleagues,^23^ to determine the sensitivity of both VSA and ISAS. All findings were assigned to one of five categories (A‐E) according to their degree of alignment with the clinical focus hypothesis in alphabetical order with decreasing accuracy findings (see Figure 2): Category A includes findings aligned on a sublobar level, Category B on a lobar level, and Category C on a hemispheric level. Category D contains findings in multiple regions on both hemispheres and Category E describes null‐result findings. In our analysis of alignment, we did not distinguish between hyper‐ and hypoperfusions since in most cases hypoperfusion was not explicitly addressed in the physicians' reports.

### 2.5 Image acquisition

Images were acquired at the Department of Nuclear Medicine at the University Hospital Bonn using an IRIX 3 head gamma camera (Philips Healthcare). Acquisition parameters were 120° head orientation, 128 matrix, 1.333 magnification, 40 steps, 3° angular step, 23 sec/step, two scans, using a low‐energy high‐resolution (LEHR) collimator. Images were reconstructed using filtered backprojection, low‐pass filter, 1.33 magnitude, 5.0 order, 0.51 cutoff, 3.5 mm slice thickness, 0.11 attenuation coefficient, 256 × 256 matrix, 2.00 zoom.

The injection was performed with an automatic MRI contrast injector (CT‐Injector Missouri TM, Ulrich Medical) and was remotely initiated and controlled from the monitoring workstation where EEG and video monitoring were used to determine seizure onset. To perform ictal SPECT, the tracer tip was covered with a lead cap to protect subjects and staff from the radioactive tracer.

### 2.6 Statistical analysis

Statistical analyses were performed with Statsmodels packages in Python 3. Regression models were run to identify which co‐factors (seizure–injection latency, Frontal and Temporal SOZ, MR‐negative individuals) moderate VSA and ISAS outcome. For this purpose, VSA and ISAS outcome was set as dependent variable and the co‐factors were included as independent variables.

For group analysis, individuals without a clinical focus hypothesis were excluded (n = 42). For the subgroup analysis of localization in temporal and frontal SOZ, we selected the two major groups of people with epilepsy with frontal and temporal clinical focus hypothesis. Seizure–injection latencies were documented in 143/146 (98%) cases, so logistic and ordinal regression was performed with missing values. The outcome‐related data shown in contingency tables were analyzed using odds ratios (ORs) and Fisher's exact test. For calculating ORs, categories A and B were merged in a subgroup of *localizing* SPECT‐results, while categories C, D, and E were merged in a subgroup of *nonlocalizing* SPECT‐results. An effect was regarded statistically significant if *P* < 0.05.

## 3 RESULTS

### 3.1 Clinical characteristics

The cohort includes 60 women and 101 men (Mean age at seizure onset ± SD: 13.9 ± 10.5 years; mean age at presurgical evaluation ± SD: 32.3 ± 12.0 years). Clinical focus hypotheses were distributed as follows: 44/161 (27%) frontal, 52/161 (32%) temporal, 6/161 (4%) parietal, 3/161 (2%) occipital, in 14/161 (9%) several foci or foci of several lobes were assumed. In 42/161 (26%) of the cohort, no focus hypothesis could be generated within the presurgical work‐up. To date, 39/161 (24%) individuals underwent epilepsy surgery (see Table 1).

**TABLE 1:** Demographics and Clinical characteristics (n = 161).

Mean age	32.3 y (±12.0 y)
Mean age of seizure onset	13.9 y (±10.5 y)
Sex	60 F / 101 M
Pathology	
On MRI	Focal cortical dysplasia 40 (25%)
	Hippocampal sclerosis 15 (9%)
	Tumor 3 (2%)
	Other 16 (10%)
Histologically confirmed in individuals underwent surgery	Focal cortical dysplasia 18 (46%)
	Type Ia 2 (11%)
	Type Ib 1 (6%)
	Type IIa 3 (15%)
	Type IIb 7 (39%)
	Not clearly specified 5 (28%)
	Hippocampal sclerosis 5 (13%)
	Wyler IV 3 (60%)
	Wyler III 1 (20%)
	Not clearly specified 1 (20%)
	Heterotopia 3 (8%)
	Tumor 4 (10%)
	Ganglioglioma 1 (25%)
	Glioma 1 (25%)
	Multinodular vacuolating tumor (Cerebrum) 1 (25%)
	Dysembryoplastic neuroepithelial tumor 1 (25%)
	Astrogliosis 6 (15%)
Clinical focus hypothesis	Frontal 44 (27%)
	Temporal 52 (32%)
	Parietal 6 (4%)
	Occipital 3 (2%)
	Multiple regions 14 (9%)
	No focus hypothesis 42 (26%)
Individuals undergoing surgery	39 (24%)
	24 (62%)
MR‐positive	100 (62%)
MR‐negative	61 (38%)
Mean seizure‐injection latency	13.56 sec (±10.4 sec)

*Note*: ±Standard deviation and percentages in brackets.

Abbreviations: F, female; M, male; MR‐negative, no potentially epileptogenic lesion identified in MRI; MR‐positive, potentially epileptogenic lesion identified in MRI; sec, seconds; y, years.

### 3.2 Differences between VSA and ISAS

**FIGURE 3:**
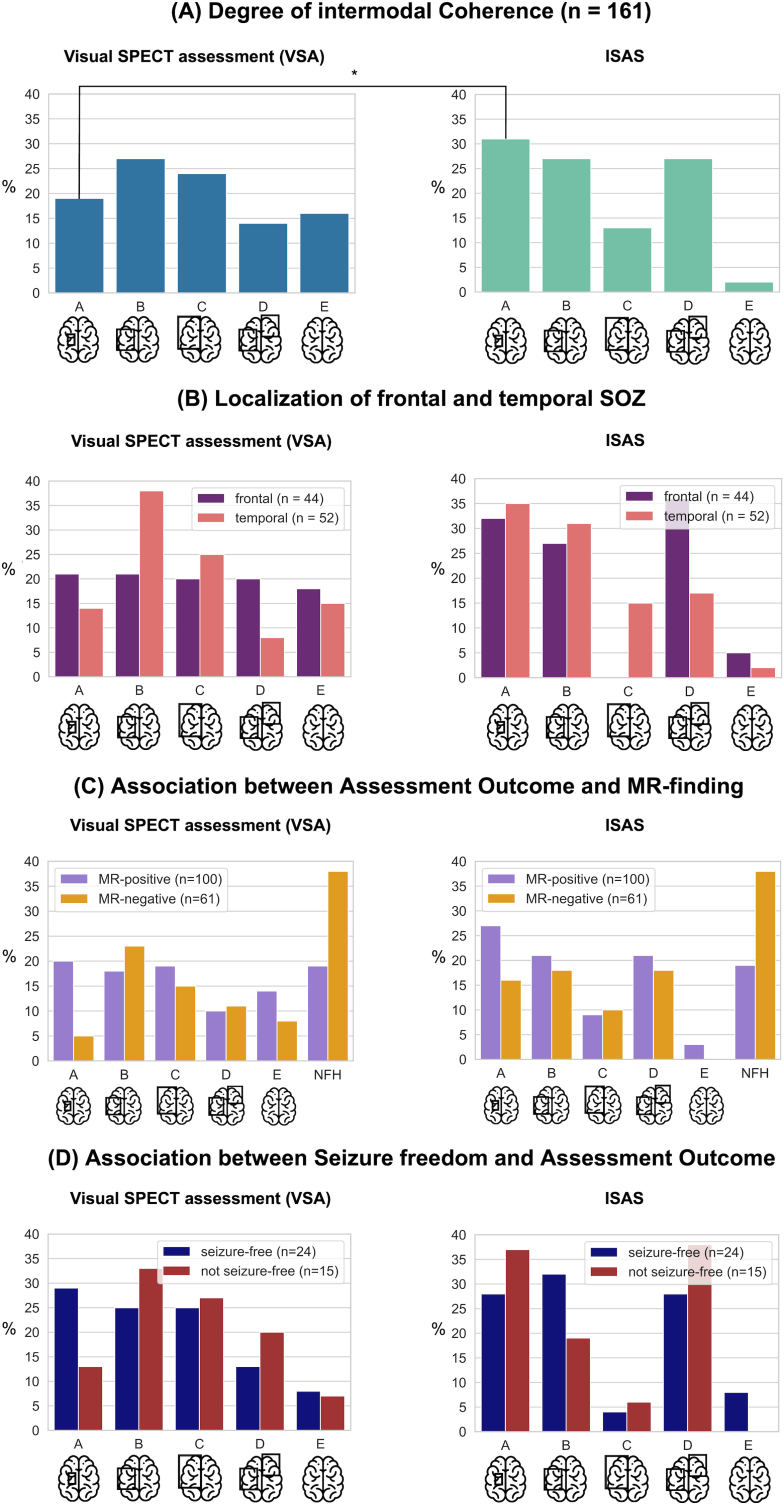
Comparisons of evaluations of ictal SPECT and of different subgroups. (A) Degree of intermodal coherence in the full cohort. The bracket marks category A (sublobar level) of the VSA and ISAS cohorts tested against categories B, C, D, E (*P* = 0.03). (B) Degree of intermodal coherence in the temporal and frontal cohort. (C) Degree of intermodal coherence of all people with and without MRI abnormalities (MR‐positive vs. MR‐negative). (D) Degree of intermodal coherence in individuals who did and who did not become seizure‐free after surgery. The x‐axis determines the classification of intermodal coherence. The y‐axis shows the percentage of individuals within the respective concordance categories (*P* < 0.05 = *).

VSA could detect the SOZ on a sublobar level (category A) in 23/119 (19%) people with epilepsy, whereas ISAS could identify the SOZ on a sublobar level in 37/119 (31%) cases. On a lobar level (category B), VSA could detect the SOZ in 32/119 (27%) cases, and ISAS could identify the SOZ in 32/119 (27%) cases. Hemispheric level findings (category C) were obtained in 28/119 (24%) people with epilepsy in the VSA cohort and in 15/119 (13%) cases in the ISAS cohort. Category D findings were obtained in 17/119 (14%) people with epilepsy in the VSA cohort and in 32/119 (27%) cases in the ISAS cohort. Nonsignificant findings (category E) were obtained in 19/119 (16%) people with epilepsy in the VSA cohort and in 3/119 (3%) cases in the ISAS cohort. The percentage of the best category A findings with ISAS was significantly higher than with VSA (OR = 1.88; 95% Cl [1.04, 3.42]; *P* = 0.03; see Figure 3A).

### 3.3 Predictors of localizing SPECT outcome

#### 3.3.1 Seizure–injection latency

The average seizure–injection latency ± SD was 13.6 ± 10.4 seconds. Using Logit Regression, neither VSA (Category C, D, E) nor ISAS findings were predicted by the seizure–injection latency (*ß*_injection–latency_ = e^−0.0065^; 95% Cl [e^−0.048^, e ^0.035^]; *P* = 0.75).

#### 3.3.2 Localization of frontal and temporal SOZ of VSA and ISAS

In both VSA and ISAS, higher concordance with the clinical focus hypothesis was obtained in individuals in whom a temporal focus was hypothesized (see Figure 3B). In the temporal VSA cohort, category A was obtained in 7/52 (13%), category B was obtained in 20/52 (38%). In the frontal VSA cohort, category A was obtained in 9/44 (20%) and category B was obtained in 9/44 (20%). In the temporal ISAS cohort, category A was obtained in 18/52 (35%), category B was obtained in 16/52 (31%). In the frontal ISAS cohort, category A was only obtained in 14/44 (32%) and category B was obtained in 12/44 (27%). However, this trend was not statistically significant (OR_VSA_ = 1.560; 95% Cl [0.693, 3.508]; *P* = 0.28. OR_ISAS_ = 1.307; 95% Cl [0.570, 2.996]; *P* = 0.52).

#### 3.3.3 Are good SPECT outcomes associated with positive MRI‐findings?

A potentially epileptogenic lesion diagnosed in MRI (MR‐positive) was associated with a high concordance category (Category A, B; see Figure 3C); however, the group difference was not statistically significant (OR_VSA_ = 1.586; 95% Cl [0.795, 3.163], *P* = 0.19. OR_ISAS_ = 1.177; 95% Cl [0.540, 2.563]; *P* = 0.68).

### 3.4 Subgroup analyses

#### 3.4.1 People who underwent epilepsy surgery (n = 39)

In 20/39 (51%) of the individuals who underwent surgery, VSA was localizing (Category A and B), while in 19/39 (49%) individuals no possible SOZ could be localized using VSA (Category C, D, E). ISAS was localizing in 24/39 (62%) individuals, while it was nonlocalizing in 15/39 (38%). 28/39 (72%) individuals became seizure‐free after surgery. With a localizing VSA (Category A, B), we found a statistical trend towards higher likelihood of postoperative seizure freedom than with a nonlocalizing VSA (OR = 1.350; 95% Cl [0.370, 4.925], *P* = 0.64). With a localizing ISAS, the likelihood of becoming seizure‐free postoperatively was about 1.2 times higher than with non‐localizing ISAS (OR = 1.1667; 95% Cl [0.3272, 4.1593]; *P* = 0.81; Figure 3D).

#### 3.4.2 People who underwent epilepsy surgery and became seizure‐free (n = 24)

**FIGURE 4:**
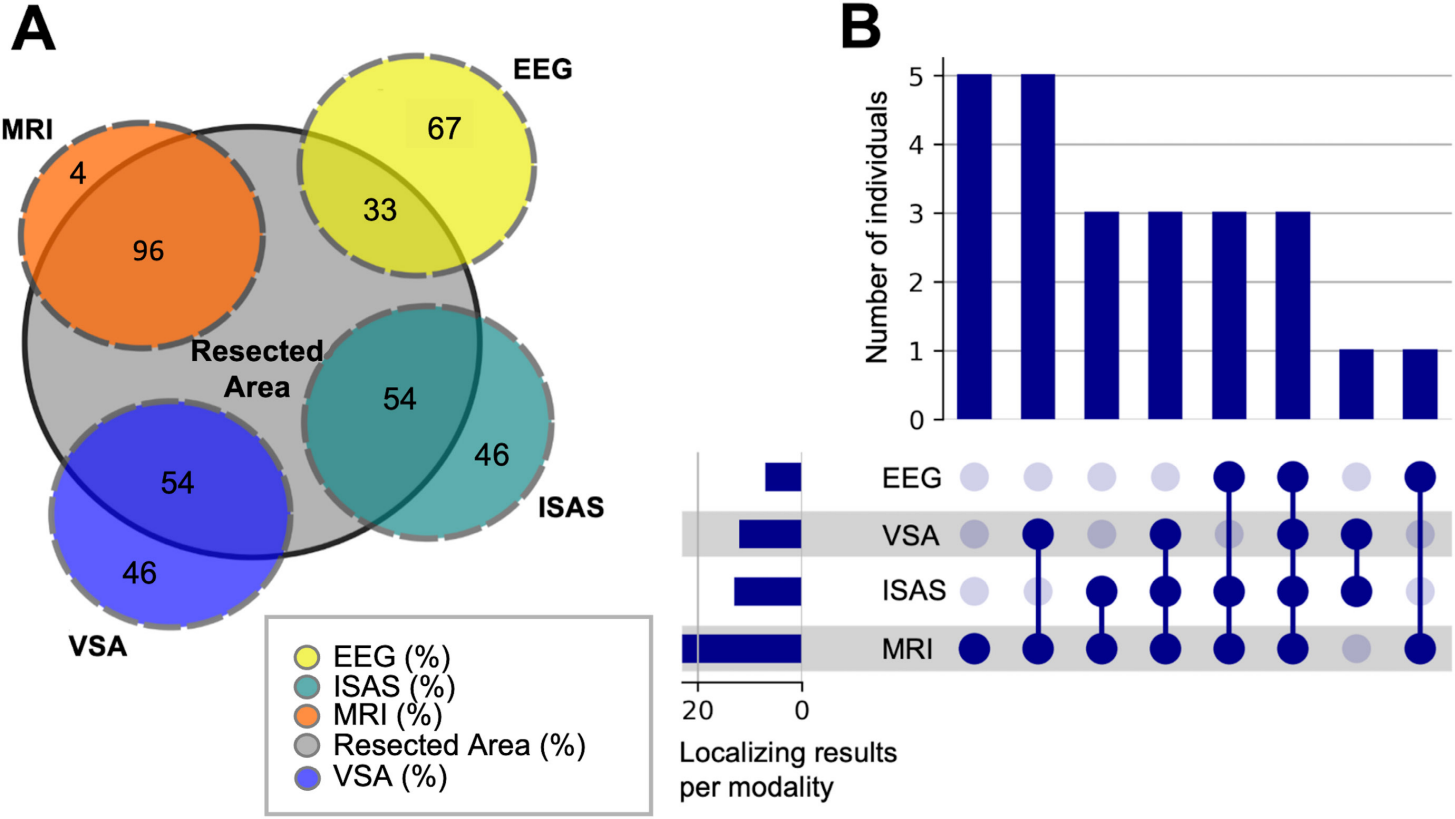
Venn diagram of diagnostic validity in seizure‐free individuals after surgery (n = 24). (A) The inner circle indicates the resected area. The four outer circles represent the four diagnostic modalities EEG, MRI, visual SPECT assessment (VSA), and ictal‐interictal SPECT Analyses by SPM (ISAS). The overlap of the modalities with the resection area indicates the percentage of cases in which the respective modality was localizing (Category A or B). The fraction not overlapping with the resection area indicates the percentage of cases in which the modality was not localizing (Category C, D or E). (B) The overlap of the presurgical modalities inter se is shown. The total size of localizing results within each modality is visualized by the lower left bar plot. Every possible intersection is represented by the lower right plot, and number of their occurrence is shown on the top bar plot. The dark blue dots show that the modality is localizing the resected area, the light blue dots show the modality is not localizing. In sum, the displayed combinations result in the 24 individuals with epilepsy.

For the subgroup of 24 seizure‐free individuals, we assessed whether presurgical modalities (interpretation of semiology, EEG, MRI, VSA, ISAS) identified the resection site correctly. Here, categories A and B were merged in a subgroup of *localizing* SPECT‐results, while categories C, D and E were merged in a subgroup of *nonlocalizing* SPECT‐results. MRI allowed the identification of the correct resection localization in 23/24 individuals (96%) of the cohort. VSA and ISAS indicated the correct resection site in 13/24 individuals (54%), whereas EEG was accurate in only 8/24 individuals (33%) (see Figure 4A). We did not only focus on each modality individually, but also on the extent to which the combination of modalities correctly predicted resection localization (see second bar of Figure 4B).

## 4 DISCUSSION

The aim of this study was to quantify the diagnostic value of ictal SPECT, either evaluated by VSA or by ISAS. Comparing both approaches, the best overlap with the presumed SOZ was increased by the factor 1.88 with ISAS than with VSA. This meets expectations and is in line with previous reports.^11^, ^12^ In cases in which preoperative assessment led to epilepsy surgery, the odds of becoming seizure‐free after surgery were higher when the SOZ was identified by ISAS or VSA preoperatively. However, Fisher's exact test did not show a significant group difference, possibly due to the relatively small group numbers. While the overall results underline the clinical value of ictal SPECT in the preoperative diagnostic evaluation for epilepsy surgery, especially when computational postprocessing was applied, the advantage on treatment decisions did not hold in all scenarios equally. In our dataset, both ISAS and VSA more likely uncovered temporal SOZs than extratemporal SOZs. This is also corroborated by previous research;^24^, ^25^, ^26^ however, the group difference was also not significant in our cohort. The sensitivity of both VSA and ISAS in our cohort was only 54% and, thus, lower than reported in the literature, where the sensitivity of VSA and postprocessing methods like ISAS and SISCOM was between 67% and 96% for temporal lobe epilepsy and 57%–66% for extratemporal epilepsy.^21^, ^24^, ^27^ However, the use of different and less finely graded outcome categories made a legitimate comparison of results difficult. Regarding seizure injection latencies, previous work showed inconsistent results. Whereas in some studies, short seizure injection latencies correlated with better VSA and ISAS outcomes,^16^, ^28^ other reports, including our current study, found no association between longer seizure injection latencies and poor VSA and ISAS outcomes.^18^, ^29^ This may detract from the dogma of immediate injection in ictal SPECT but is more likely due to the small number of cases with prolonged infusion latencies (>45 seconds) in our patient population. In the general comparison, VSA and ISAS outperformed EEG in its ability to localize the SOZ but are less sensitive than MRI based on conventional radiological readings. It is important to note that the real diagnostic power of EEG extends far beyond its ability to localize a SOZ. One possible reason for the high concordance between MRI and VSA findings in our data could be the fact that the results of radiological MRI readings were available to the VSA raters. Hence, these modalities could not be considered completely independent of each other (“data leakage”). ISAS, which provides statistically determined output clusters, is not as vulnerable to observer bias. In our cohort, a positive MRI finding indicated a higher chance of a favorable surgical outcome. In individuals with epilepsy and with MRI‐negative findings, often no clinical focus hypothesis could be generated, so it remains questionable whether ictal SPECT brings an added value regarding a decision for or against surgical therapy. It is striking how 61 MR‐negative individuals received VSA and ISAS, but of these, only five underwent surgery and no one became seizure‐free. Of 100 MR‐positive individuals, 39 underwent surgery and 24 became seizure‐free. The value of ictal SPECT may lie in the confirmation or rejection of the focus hypothesis in MR‐positive cases and less in the localization of a SOZ in MR‐negative ones.

### 4.1 Limitations

Four aspects limit the power of this study. First, VSA or ISAS is only one of many modalities considered in the presurgical evaluation of epilepsy patients, and a clinical focal hypothesis was generated from all available modalities in practice. Therefore, in a retrospective analysis, it was not possible to determine the individual weights of each modality or how much they influenced each other in generating a clinical focal hypothesis. This sets limits to the validity of the “ground truth” in people who did not undergo surgery.

Second, our analysis is biased by the way the ground truth of the SOZ was defined: In people with epilepsy who did not undergo surgery, we could only compare VSA and ISAS findings with unconfirmed clinical focus hypothesis, whereas in the people who underwent surgery and became seizure‐free, the resection site/surgery outcome could be used as a valid ground truth. Third, our study suffered from the challenges of retrospective analysis. Not all data, such as seizure–injection latencies, were documented in all cases (143/146, 98%), so logistic and ordinal regression was performed with missing values.

Fourth and finally, in most VSA and ISAS reports, hypoperfusions were not addressed so that we could not consider hyperperfusion and hypoperfusion separately in our analysis. This is particularly unfortunate, as the diagnostic value of postictal hypoperfusion in the literature remains unclear.^11^, ^12^ In view of the short injection latencies, our collective may not be suitable for a study on postictal hypoperfusion anyway.

## 5 CONCLUSION

Our results demonstrate the usefulness of ictal SPECT in the presurgical evaluation for epilepsy surgery, especially when combined with computational postprocessing pipelines. The diagnostic power of ictal SPECT, however, does not equally apply in all scenarios. To further investigate the predictive power of ISAS and VSA and to overcome the limitations of a single‐center, retrospective experience, a prospective multicenter study is encouraged to improve the quality of presurgical evaluation for epilepsy surgery. Even more critical, the postprocessing methods for ictal SPECT are nearly two decades old. Although there are approaches to combine different imaging modalities such as MRI and SPECT (SISCOM, STATISCOM, ISAS) or combination of ictal SPECT and FDG‐PET (PISCOM), which could minimize radiation exposure by omitting an interictal SPECT image and increase the sensitivity to detect a SOZ,^30^ these possibilities are rarely used or further developed. They do not exploit the vast possibilities available through the advent of more advanced medical image processing, including approaches based on artificial intelligence. By demonstrating the diagnostic value of VSA and ISAS, this study and others set the bar, which has to be overcome by any future approaches.

## AUTHOR CONTRIBUTIONS

FS, FB, TBauer, HV, ME, RS and TR contributed to the conception and design of the study. FCG, RvW, TBaumgartner, AR, VB, TvO collected data. FS organized the database. FS and FB performed the statistical analysis. FS wrote the first draft of the manuscript. FB, FCG, TBauer, and TR gave administrative, technical, or material support. TR supervised the study. All authors contributed to manuscript revision, read, and approved the submitted version.

## FUNDING INFORMATION

FS was supported by the BONFOR research commission (Grants Nr. April 30, 2021) of the medical faculty of the University of Bonn.

## CONFLICT OF INTEREST

FS, FB, FCG, and TBauer have no conflict of interest to disclose. RvW has received travel support, fees as speaker or for serving on the advisory board from Angelini, Apocare, Arvelle, Cerbomed, Desitin, Eisai, GW pharmaceuticals‐JAZZ pharma and UCB Pharma. These activities were not related to the content of this manuscript. TBaumgartner and AR have no conflict of interest to disclose. VB has received fees for serving as clinical consultant from Brainlab AG. These activities were not related to the content of this manuscript. TvO and HV have no conflict of interest to disclose. RS has received fees as speaker or for serving on the advisory board from Angelini, Arvelle, Bial, Desitin, Eisai, Janssen‐Cilag GmbH, LivaNova, Novartis, Precisis GmbH, UCB Pharma, UnEEG and Zogenix. These activities were not related to the content of this manuscript. TR declares that the research was conducted in the absence of any commercial or financial relationships that could be constructed as a potential conflict of interest. The results were presented in a poster at the DGFE 2022 in Leipzig and at the OHBM 2022 in Glasgow.

## ETHICAL APPROVAL

We confirm that we have read the Journal's position on issues involved in ethical publication and affirm that this report is consistent with those guidelines. The study was approved by the IRB of the University Hospital Bonn.

## Data Availability

The data that support the findings of this study are available on request from the corresponding author. The data are not publicly available as they contain information that could compromise the privacy of research participants.
